# Application and Validation of the Tricuspid Annular Plane Systolic Excursion/Systolic Pulmonary Artery Pressure Ratio in Patients with Ischemic and Non-Ischemic Cardiomyopathy

**DOI:** 10.3390/diagnostics11122188

**Published:** 2021-11-24

**Authors:** Stanislav Keranov, Saskia Haen, Julia Vietheer, Wiebke Rutsatz, Jan-Sebastian Wolter, Steffen D. Kriechbaum, Beatrice von Jeinsen, Pascal Bauer, Khodr Tello, Manuel Richter, Oliver Dörr, Andreas J. Rieth, Holger Nef, Christian W. Hamm, Christoph Liebetrau, Andreas Rolf, Till Keller

**Affiliations:** 1 Department of Internal Medicine I, Cardiology and Angiology, Justus-Liebig-University Giessen, 35392 Giessen, Germany; saskia.m.haen@med.uni-giessen.de (S.H.); wiebke.rutsatz@googlemail.com (W.R.); pascal.bauer@innere.med.uni-giessen.de (P.B.); oliver.doerr@innere.med.uni-giessen.de (O.D.); Holger.Nef@innere.med.uni-giessen.de (H.N.); Christian.Hamm@innere.med.uni-giessen.de (C.W.H.); till.keller@med.uni-giessen.de (T.K.); 2 DZHK (German Center for Cardiovascular Research), Partner Site RheinMain, 61231 Bad Nauheim, Germany; s.kriechbaum@kerckhoff-klinik.de (S.D.K.); c.liebetrau@ccb.de (C.L.); 3 Kerckhoff Heart and Thorax Center, Department of Cardiology, 61231 Bad Nauheim, Germany; j.vietheer@kerckhoff-klinik.de (J.V.); j.wolter@kerckhoff-klinik.de (J.-S.W.); b.vonjeinsen@kerckhoff-klinik.de (B.v.J.); a.rieth@kerckhoff-klinik.de (A.J.R.); a.rolf@kerckhoff-klinik.de (A.R.); 4 Department of Internal Medicine, Justus-Liebig-University Giessen, Universities of Giessen and Marburg Lung Center (UGMLC), Member of the German Center for Lung Research (DZL), 35392 Giessen, Germany; Khodr.Tello@innere.med.uni-giessen.de (K.T.); Manuel.Richter@innere.med.uni-giessen.de (M.R.); 5 Cardioangiological Center Bethanien (CCB), 60389 Frankfurt, Germany

**Keywords:** TAPSE/PASP, RVEF, MRI, T1 mapping, RV remodeling, LV remodeling

## Abstract

The main aim of this study was to assess the prognostic utility of TAPSE/PASP as an echocardiographic parameter of maladaptive RV remodeling in cardiomyopathy patients using cardiac magnetic resonance (CMR) imaging. Furthermore, we sought to compare TAPSE/PASP to TAPSE. The association of the echocardiographic parameters TAPSE/PASP and TAPSE with CMR parameters of RV and LV remodeling was evaluated in 111 patients with ischemic and non-ischemic cardiomyopathy and cut-off values for maladaptive RV remodeling were defined. In a second step, the prognostic value of TAPSE/PASP and its cut-off value were analyzed regarding mortality in a validation cohort consisting of 221 patients with ischemic and non-ischemic cardiomyopathy. A low TAPSE/PASP (<0.38 mm/mmHg) and TAPSE (<16 mm) were associated with a lower RVEF and a long-axis RV global longitudinal strain (GLS) as well as higher RVESVI, RVEDVI and NT-proBNP. A low TAPSE/PASP, but not TAPSE, was associated with a lower LVEF and long-axis LV GLS, and a higher LVESVI, LVEDVI and T1 relaxation time at the interventricular septum and the RV insertion points. Furthermore, in the validation cohort, low TAPSE/PASP was associated with a higher mortality and TAPSE/PASP was an independent predictor of mortality. TAPSE/PASP is a predictor of maladaptive RV and LV remodeling associated with poor outcomes in cardiomyopathy patients.

## 1. Introduction

RV dysfunction is an important prognostic factor in many cardiopulmonary disorders including left-sided heart failure [1,2]. Many patients with ischemic and non-ischemic cardiomyopathy develop maladaptive right ventricular (RV) remodeling that is associated with dilation, sarcomere changes, fibrosis, capillary rarefication and metabolic disorders in the RV myocardium [3,4]. These changes lead to progressive RV dysfunction and eventually to right heart failure. 

The current, non-invasive gold standard for the assessment of RV structure, dimensions and function is cardiac magnetic resonance (CMR) imaging. It provides high accuracy and reproducibility [5,6]. MRI-derived indices such as the right ventricular ejection fraction (RVEF), the right ventricular end-diastolic volume index (RVEDVI) and the right ventricular end-systolic volume index (RVESVI) are predictors of adverse outcomes in various cardiopulmonary disorders [5,6,7,8], including ischemic and non-ischemic cardiomyopathy [4,9,10,11,12,13]. However, CMR is an expensive and time-consuming diagnostic modality that is also limited in its availability.

The TAPSE/PASP ratio is a novel echocardiographic parameter that has been validated as a predictor of major adverse events in patients with heart failure and reduced left ventricular (LV) ejection fraction (HFrEF), as well as in heart failure patients with preserved LV ejection fraction (HFpEF) [14,15,16]. However, valid evidence on its association with CMR parameters of RV structure and function is still scarce. Further research is also needed to analyze its potential advantages when compared to established echocardiographic parameters such as TAPSE.

Hence, the aim of this study was to analyze and compare the association of TAPSE/PASP and TAPSE with the CMR parameters of RV and LV remodeling in a group of patients with ischemic and non-ischemic cardiomyopathy. Furthermore, we sought to identify a TAPSE/PASP cut-off value associated with the relevant RV maladaptation and to validate its association with mortality in another independent cohort of patients with ischemic and non-ischemic cardiomyopathy.

## 2. Materials and Methods

### 2.1. Study Cohorts

The present analysis uses patients of two independent, ongoing registry studies. Both cross-sectional studies (BioCVI and BioProspective) are part of the Kerckhoff Biomarker Registry (BioReg). Participation in the registry, including both study cohorts, was voluntary; all patients gave written informed consent and the studies were approved by the local ethics committee. 

Firstly, as the imaging cohort, patients were recruited from the BioCVI study [17]. Here, adult patients (>18 years old) with a clinical indication for CMR are enrolled. For this evaluation, 111 patients enrolled between May 2016 and May 2020 were selected based on the following criteria: diagnosis ischemic or non-ischemic cardiomyopathy and availability of an 2D echocardiographic evaluation with valid TAPSE and PASP measurements.

This imaging cohort was used to evaluate the association of TAPSE, PASP and TAPSE/PASP with CMR parameters of maladaptation known to be associated with prognosis such as RVEF, RVESVI, RVEDVI, RV Strain and T1 mapping.

Secondly, as the clinical validation cohort, patients were recruited from the BioProspective study [18]. Adult patients (>18 years old) with a clinical indication for invasive coronary angiography were enrolled. For the present analyses, 609 patients enrolled between August 2010 and March 2020 were selected based on the following criteria: diagnosis of ischemic or non-ischemic cardiomyopathy and having available data on all-cause mortality. 

TAPSE measurements were available in 321 patients, PASP was measured in 306 patients and 221 patients had valid TAPSE/PASP values. Hence, this was the validation cohort used for further analyses. A total of 89 of these 221 patients were known to be deceased by July 2020, and median time between enrollment and the end day of the follow-up was 6.2 years.

### 2.2. Transthoracic Echocardiography 

All evaluated patients underwent transthoracic two-dimensional echocardiography, using commercially-available ultrasound systems (EPIQ 7, Philips Ultrasound Systems, Koninklijke Philips N.V., Amsterdam, The Netherlands; E9 and S5, General Electric, Boston, MA, USA) as part of the clinical diagnostic workup. Left and right ventricular assessment was performed as recommended by the recent guidelines. TAPSE was obtained by placing an M-mode cursor through the lateral tricuspid annulus in the apical four-chamber view. TAPSE was measured as the peak excursion of the tricuspid annulus from the end of diastole to the end of systole. Pulmonary artery systolic pressure (PASP) was obtained as the sum of the pressure gradient between the right atrium and the right ventricle, acquired from the TR jet peak velocity and the right atrial pressure (RAP) estimation, which was based on the size of the inferior vena cava and its collapsibility. In 20 BioCVI and 80 BioProspective patients, TAPSE were also measured using a commercially-available post-processing software (TomTec, Philips Ultrasound Systems, Koninklijke Philips N.V., Amsterdam, The Netherlands).

### 2.3. Cardiac MRI

Imaging was performed using a 3 Tesla scanner system (Magnetom Skyra, Siemens Healthcare, Forchheim, Germany) with a dedicated CMR protocol containing axial, coronal and sagittal thoracic survey images, CINE sequences, steady-state-free precession sequences (SSFP) in 2-, 3-, and 4-chamber views. Typical sequence parameters were as follows: a TE/TR/flip-angle of 1.7 ms/3.4 ms/60° and aspatial resolution of 1.8 × 1.8 × 8 mm.

The T1 mapping was performed using a modified Look-Locker Imaging (TE/TR/flip-angle: 1.64 ms/3.3 ms/50°, acquired voxel size 1.8 × 1.8 × 8 mm, phase encoding steps *n* = 166, 6/8 half scan, 11 images corresponding to three different inversion times using a nonselective 180° prepulse in an algorithm of *n*-images/*n*-beats 3 (2) 3 (2) 5 MOLLI scheme), as published previously [19].

The T1 times were obtained for myocardium in regions of interest (ROI) at basal, midventricular and apical SA sections. Hence, nine ROIs were manually drawn in the septum and the upper- and lower-septal right ventricular insertion points (RVIP) in each SA section. All ROIs were drawn only on the compact myocardium and did not encompass the myocardial borders. The post-processing was performed with the syngo.via software package (Siemens Syngo, Siemens Healthcare, Forchheim, Germany). Post-processing of the right and left ventricular volumes was performed using the cvi42 software package (circle cardiovascular imaging, Calgary, AB, Canada). Endo- and epicardial borders were defined using an artificial intelligence-based algorithm and carefully corrected by two experienced readers.

### 2.4. Statistical Analysis

Continuous variables are presented as mean ± standard deviation (SD) or median with 25th to 75th interquartile range, (IQR), as appropriate. Categorical variables are expressed as numbers and percentages. Parametric distribution was assessed with the Shapiro–Wilk test. Normally-distributed continuous variables were compared using the Welch two-sample *t*-test and a one-way analysis of variance with the Bonferoni post-hoc test. The Mann-Whitney U test and the Kruskal-Wallis test with Dunnet’s post-hoc test were used for the non-normally distributed continuous variables. The receiver operating characteristic (ROC) curve analysis was performed to assess the predictive value of TAPSE and TAPSE/PASP regarding RVEF < 35%, RVEDVI < 100 mL/m^2^ and NT-proBNP < 1000 pg/mL. A two-tailed *p* value < 0.05 was considered to indicate statistical significance. Statistical analysis was performed using R software and SPSS Version 25.0.0 (SPSS Inc., Chicago, IL, USA).

## 3. Results

### 3.1. Baseline Characteristics

The baseline characteristics of the imaging cohort (*n* = 111) are shown in Table 1. The median age was 69 years (IQR 60; 76), 30% of the patients were female. Based on the CMR measures LVEF and RVEF were reduced with a median of 35% (IQR 24; 50) and of 40% (IQR 27; 51), respectively. 

#### 3.1.1. Associations of the Echocardiographic TAPSE/PASP Ratio and CMR

The echocardiographic parameters TAPSE and TAPSE/PASP were both moderately correlated with the CMR volume parameters RVEF, RVEDVI and RVESVI. Further, TAPSE/PASP significantly correlated with LVEF, LVEDVI, LVESVI, LA area and LV mass, whereas no significant correlations between TAPSE and all LV CMR parameters were observed. 

CMR strain analyses representing subtle functional CMR parameters showed significant correlations of TAPSE/PASP with RV and LV long-axis global longitudinal strain. TAPSE alone was also correlated with RV and LV long-axis global longitudinal strain.

To reflect myocardial structure, analyses of T1 mapping were performed and showed a significant correlation of TAPSE/PASP with septum T1 time and the T1 determined in the lower and upper RV insertion points. TAPSE did not correlate with any T1 time.

Beyond CMR parameters, the biomarker NT-proBNP, an established, easily-accessible marker that represents hemodynamic changes, was evaluated. TAPSE/PASP showed a strong correlation with NT-pro-BNP that was significantly higher than the correlation of TAPSE with NT-pro-BNP. 

Detailed results of the correlation analyses of TAPSE and TAPSE/PASP with CMR parameters and cardiac biomarkers are presented in Table 2. 

**Figure 1:**
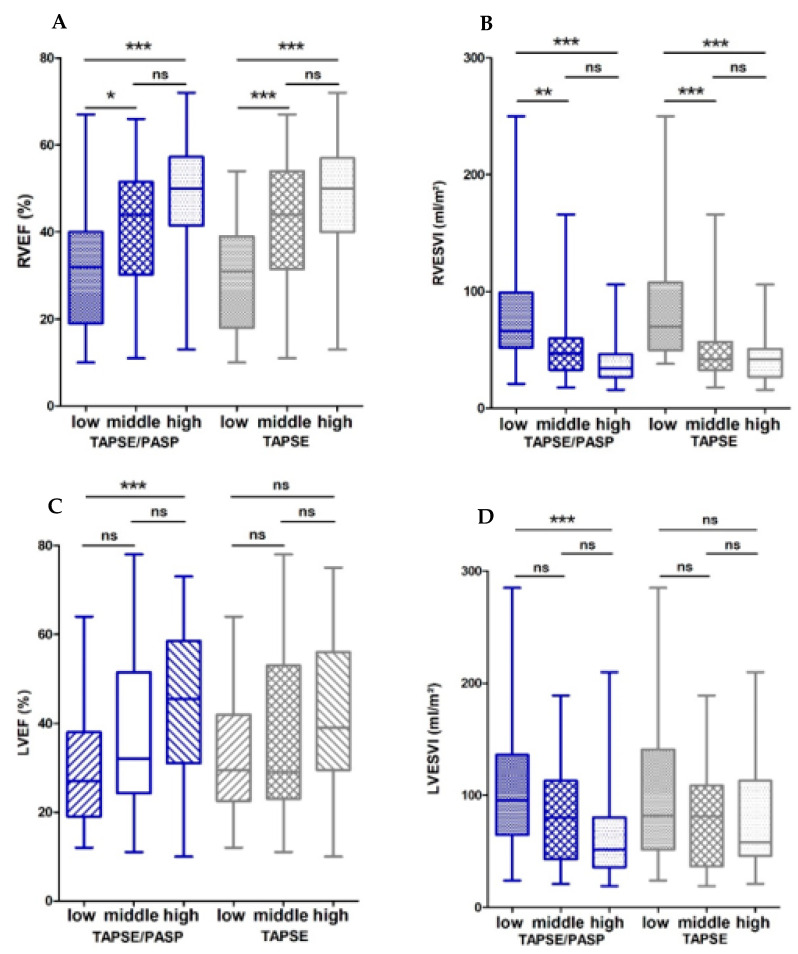
Box plots showing CMR characteristics of TAPSE/PASP and TAPSE tertiles. Shown are values for (**A**) RVEF, (**B**) RVESVI, (**C**) LVEF and (**D**) LVESVI divided according to TAPSE/PASP and TAPSE tertiles. Boxes represent median with IQR. ns, not significant, * *p* < 0.05, ** *p* < 0.01, *** *p* < 0.001.

To simultaneously meet the requirements of an easy clinical application and to consider the continuous nature of the TAPSE/PASP ratio, we further performed analyses based on the TAPSE/PASP tertiles leading to the groups low, intermediate and high (low: <0.38 mm/mmHg, intermediate: 0.38–0.63 mm/mmHg, high: >0.63 mm/mmHg). In terms of clinical characteristics, there were no significant differences between these three groups (Supplementary Table S1). However, TAPSE/PASP groups showed substantial differences regarding CMR parameters. Patients in the low TAPSE/PASP tertile had a lower median RVEF of 32% compared to 44% (*p* < 0.05) in the intermediate and 50% (*p* < 0.001) in the high tertile (Figure 1A). Conversely, the CMR-derived parameters RVESVI and RVEDVI were higher in the low TAPSE/PASP tertile than in the other two tertiles (Figure 1B and Supplementary Figure S1A). In terms of LV structure and function, LVEF was lower, with correspondingly higher LVESVI and LVEDVI observed in the low tertile compared to the high tertile (Figure 1C,D, Supplementary Figure S1B).

**Figure 2:**
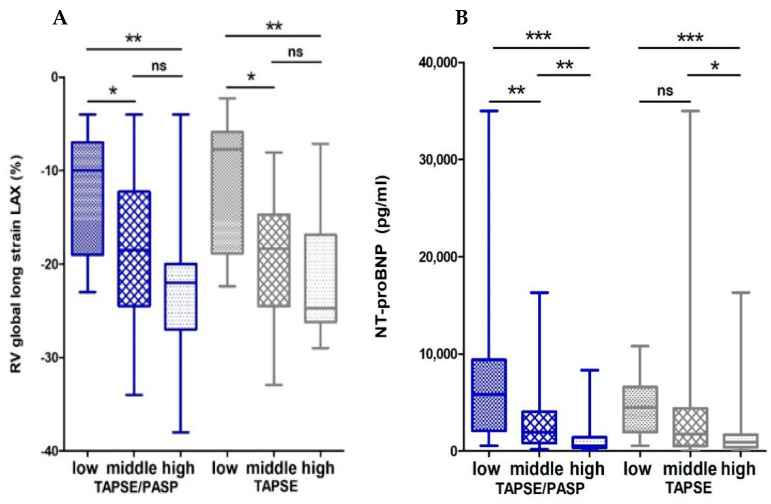
Box plots showing RV global longitudinal strain and NT-proBNP values of TAPSE/PASP and TAPSE tertiles. Shown are values for (**A**) long axis RV global longitudinal strain and (**B**) NT-proBNPdivided according to TAPSE/PASP and TAPSE tertiles. Boxes represent median with IQR. ns, not significant, * *p* < 0.05, ** *p* < 0.01, *** *p* < 0.001.

**Figure 3:**
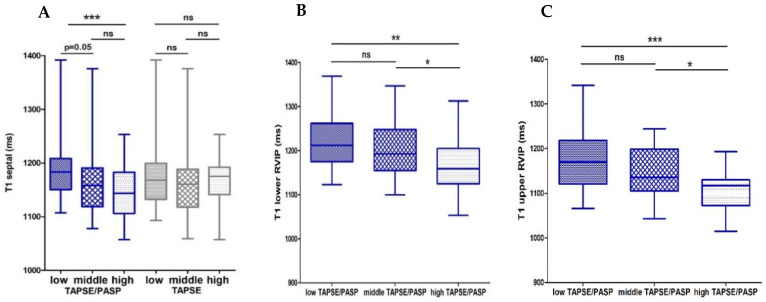
Box plots showing T1 relaxation time values of TAPSE/PASP and TAPSE tertiles. Those shown are values for (**A**) T1 septal divided according to TAPSE/PASP and TAPSE tertiles, (**B**) T1 lower right ventricular insertion point (RVIP) and (**C**) upper RVIP divided according to TAPSE/PASP tertiles. Boxes represent median with IQR. ns, not significant, * *p* < 0.05, ** *p* < 0.01, *** *p* < 0.001.

The RV function reflected by RV long-axis global longitudinal strain was reduced in the lowest TAPSE/PASP tertile (Figure 2A). Native septal T1 time and native T1 time in the lower and upper RVIP was shortest in patients in the highest TAPSE/PASP tertile (Figure 3A–C).

NT-proBNP showed also differences in concentrations according to TAPSE/PASP tertiles with the lowest tertile having the highest NT-proBNP values (Figure 2B).

To present the data of the TAPSE/PASP ratio in the context of the established echocardiographic RV parameter TAPSE alone, we also analyzed the cohort according to TAPSE tertiles (low: <16 mm, intermediate: 16–20 mm, high: >0.63). These analyses showed similar results in terms of right ventricular function and structure as compared to TAPSE/PASP tertiles. RVEF was reduced and the volumes RVEDVI and RVESVI were higher in the lowest TAPSE tertile (Figure 1A,B and Supplementary Figure S1A). Global long axis RV longitudinal strain and NT-proBNP levels were also highest in the low TAPSE tertile (Figure 2A,B). However, no differences between the TAPSE tertiles and LVEF, LVEDVI, LVESVI (Figure 1C,D and Supplementary Figure S1B) or T1 times (Supplementary Figure S2A,B) were observed.

#### 3.1.2. Echocardiographic TAPSE/PASP to Predict Maladaptive RV

**Figure 4:**
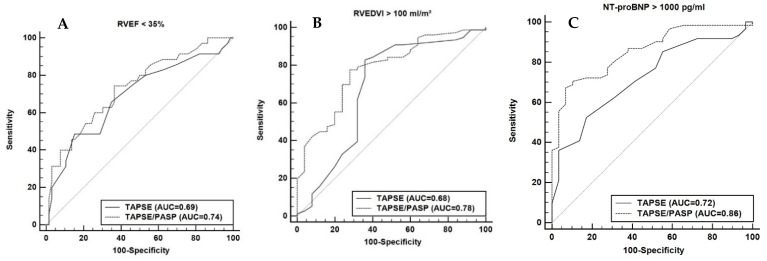
Receiver-operating characteristics curve showing the predictive power of TAPSE/PASP and TAPSE for (**A**) RVEF < 35%, (**B**) RVEDVI > 100 mL/m^2^ and (**C**) NTpro-BNP > 1000 pg/mL.

In the ROC analysis, TAPSE/PASP and TAPSE showed good predictive power for RVEF < 35%, with an AUC of 0.74 for TAPSE/PASP and of 0.69 for TAPSE alone (*p* for AUC_TAPSE/PASP_ vs. AUC_TAPSE_ = 0.2, Figure 4A). However, to predict a RVEDVI > 100 mL/m^2^, TAPSE/PASP was superior to TAPSE alone with an AUC of 0.78 vs. 0.68 (*p* for AUC_TAPSE/PASP_ vs. AUC_TAPSE_ = 0.048, Figure 4B). Further, TAPSE/PASP was more strongly associated with an elevated NT-proBNP (>1000pg/mL) compared to TAPSE alone with AUCs of 0.86 and 0.72 (p for AUC_TAPSE/PASP_ vs. AUC_TAPSE_ = 0.002, Figure 4C).

#### 3.1.3. Echocardiographic TAPSE/PASP as Predictor of Mortality

**Figure 5:**
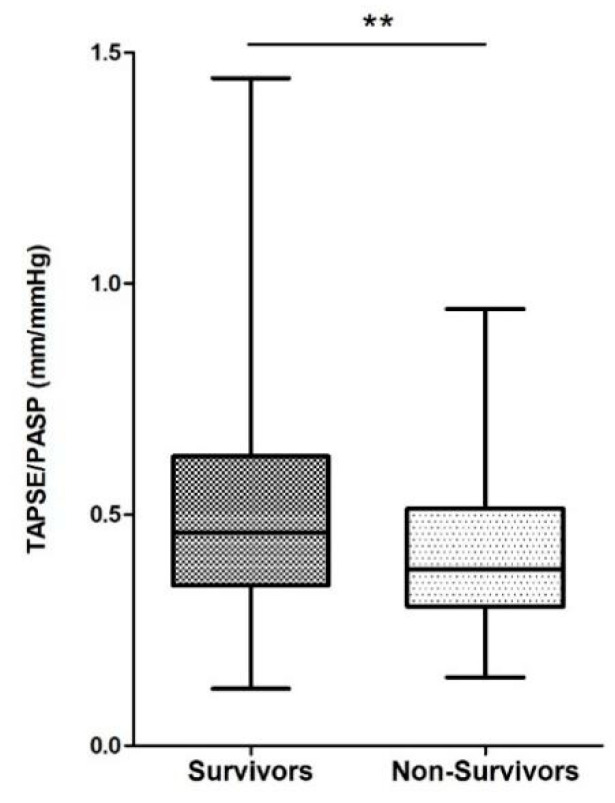
Box plots comparing TAPSE/PASP values in survivors and non-survivors. Boxes represent median with IQR. ** *p* < 0.01.

The baseline characteristics of the validation cohort (*n* = 221) are shown as Table 3. The median age was 72 years; 62% had CAD and 38% had prior PCI. All-cause mortality was 40% (*n* = 89). The TASPE/PASP ratio was lower in non-survivors compared to survivors (*p* = 0.002, Figure 5) with a correspondingly higher PASP (*p* = 0.002) in non-survivors. In terms of the established echocardiographic parameter TAPSE, no significant difference, only a trend, between survivors and non-survivors was observed (*p* = 0.12). 

The use of the TAPSE/PASP threshold between the low and the intermediate tertile of the initial imaging cohort as a prognostic cut-off (<0.38 mm/mmHg) was also associated with all-cause mortality (*p* = 0.01) in the univariate analysis. Apart from TAPSE/PASP, higher LVEDd, higher age, lower LVEF, lower eGFR, prior PCI and diabetes were also associated with all-cause mortality in the univariate analysis and, therefore, were analyzed together with either TAPSE/PASP or PASP as predictors of mortality in a multivariable regression model. Here, only TAPSE/PASP, LVEDd and prior PCI remained independent predictors of mortality (Supplementary Table S2).

## 4. Discussion

To the best of our knowledge, the present study is the first to assess the association of the echocardiographic parameter TAPSE/PASP with CMR parameters of maladaptive RV and LV remodeling in patients with ischemic and non-ischemic cardiomyopathy. 

The main findings of the study are:

(1) Low TAPSE/PASP (cut-off 0.38 mm/mmHg) and a low TAPSE (cut-off 16 mm) are both associated with RV dysfunction, RV dilation and higher NT-proBNP levels;
(2) TAPSE/PASP, but not TAPSE alone, shows an association with LV dysfunction, LV dilation and fibrosis in the ventricular septum and RV insertion points;
(3) TAPSE/PASP < 0.38 mm/mmHg is associated with a significantly increased mortality and TAPSE/PASP is an independent predictor of mortality.

By providing high spatial resolution and 3D volumetric data, CMR allows accurate quantification of cardiac function and dimensions. This is especially important in RV assessment because of its distinctive geometry [20,21]. Furthermore, CMR measurements in healthy subjects as well as in patients with various cardiovascular disorders show high reproducibility [5,6,11,22].

Existing evidence shows that CMR assessment of RV function and structure does provide valid information for the risk stratification of patients with ischemic and non-ischemic cardiomyopathy. In a study of 250 patients with non-ischemic dilated cardiomyopathy, a CMR derived RVEF of < 45% was an independent predictor of cardiovascular death or transplantation [11]. Further, volumetric RV indices such as RVEDVI and RVESVI were also associated with mortality in this study. These data are supported by other studies where RVEF was also a predictor of mortality in patients with ischemic cardiomyopathy [10,23]. Another large CMR cohort of 588 patients with ischemic cardiomyopathy also showed that a reduced RVEF of < 35% was associated with a higher mortality [12]. Several studies in patients with pulmonary hypertension (PH) further showed an association of RV volumes and RVEF with adverse outcomes [7,24,25,26]. 

These robust and available data on CMR, which is an established method to stratify RV-mediated risk, motivated us to use CMR as a reference method for the assessment of prognostically-relevant RV and LV maladaptive remodeling via echocardiographic imaging parameters. 

The measurement of TAPSE during echocardiography is already an established parameter to estimate RV function. It is a simple and cost-effective method for the measurement of RV systolic function and a threshold of 16 mm is recommended by the current guidelines [27] as a prognostic cut-off for RV dysfunction.

The TAPSE/PASP ratio was proposed as a novel echocardiographic parameter that is not as load-dependent as TAPSE; thus, it might define RV-PA coupling in a non-invasive way [15]. Measuring Ees/Ea in pressure-volume loops as a surrogate for RV-PA coupling is the current, invasive gold standard for the assessment of the RV function in relation to pulmonary afterload and the transition to right heart failure [8]. TAPSE/PASP correlates well with Ees/Ea in patients with HFpEF [16] and HFrEF [28]. It was also associated with higher mortality in HFpEF and in HFrEF patients [14,28,29]. Studies in patients with PH also showed an independent association of TAPSE/PASP with Ees/Ea, suggesting that TAPSE/PASP could be a non-invasive parameter of RV-PA coupling [30,31].

However, a recent study showed a good correlation for both TAPSE and TAPSE/PASP with Ees/Eea in HFrEF patients and both parameters were independent predictors of mortality [28]. These findings raise the question about the additional value of relating TAPSE to PASP compared to the use of TAPSE alone.

The novel aspect of our study is that it analyzes the role of TAPSE and TAPSE/PASP for RV and LV remodeling using CMR imaging. The results from our imaging cohort showed a similar correlation of TAPSE and TAPSE/PASP with RVEF, RVESVI and RVEDVI. Tertile analysis further showed that TAPSE/PASP < 0.38 mm/mmHg and TAPSE < 16 mm are both associated with significantly worse RVEF and RV long-axis global longitudinal strain and RV dilation, making these thresholds the potential cut-off values for RV maladaptation in patients with cardiomyopathies. Both parameters were also good predictors of RVEF < 35% with TAPSE/PASP better predicting a mild dilation in the ROC analysis. Hence, our study supports that TAPSE/PASP and TAPSE might provide comparable information in assessing RV systolic dysfunction and dilation via standard 2D echocardiography. 

Beyond systolic RV dysfunction and dilation, the amount of fibrosis also appears to be an important prognostic sign of RV maladaptation. Histological and cardiac imaging studies show a significant increase in RV fibrosis in patients with PH and right heart failure as compared to healthy controls [32,33,34]. RV fibrosis is associated with impaired diastolic RV function and an unfavorable prognosis in PAH patients [35,36].

Our study could not show a correlation of TAPSE alone with CMR derived parameters of fibrosis, whereas lower TAPSE/PASP showed a significant association with increased fibrosis in the septum and at the RV insertion points as measured by T1 relaxation time. Several studies already have shown that elevated T1 relaxation times at the insertion points could be early markers of disease progression in PH that occur before the deterioration of RV function [37,38]. Insertion points are particularly exposed to mechanical stress in the settings of RV pressure overload, which leads to early remodeling in these regions, depending on the degree of afterload increase [38]. Accordingly, it was described that T1 relaxation times at the insertion points correlated to afterload parameters such as mPAP and PVR [39]. This correlation of T1 relaxation times to RV afterload could be a possible explanation for the better predictive value of TAPSE/PASP for RV fibrosis when compared to TAPSE. Therefore, putting TAPSE in relation to PASP might allow a better correlation to RV afterload than TAPSE alone. This idea is supported by the invasive PV loop measurements that showed a stronger correlation of TAPSE/PASP to parameters of afterload and RV diastolic dysfunction, compared to TAPSE alone in HFrEF patients [28]. 

The most common cause for secondary PH in patients with LV pathologies is an elevated filling pressure due to functional and structural LV alterations [40]. Therefore, the relation of TAPSE/PASP to RV afterload could also, in part, explain the observed association with the parameters of systolic and diastolic LV function and the structure in our imaging cohort, which was not present in terms of TAPSE. This hypothesis is also supported by the stronger correlation of TAPSE/PASP to NT-proBNP and LA size, which are both associated with LV filling pressure.

Hence, compared to previous studies, our analysis provides a detailed assessment of the associations between TAPSE, TAPSE/PASP and important parameters of RV and LV remodeling. In our second cohort, TAPSE/PASP was an independent predictor for mortality. The defined TAPSE/PASP cut-off (0.38 mm/mmHg) for prognostically-relevant RV dysfunction that stemmed from the imaging cohort was also associated with an increased mortality in cardiomyopathy patients in this independent cohort. Interestingly, TAPSE/PASP below a threshold of 0.38 mm/mmHg was also identified as a mortality cut-off in the study comparing TAPSE/PASP with invasive PV loop measurements in HFrEF patients [28]. In HFpEF patients, a TAPSE/PASP cut-off of 0.35 mm/mmHg was described as an independent predictor of the adverse outcomes in a study comparing TAPSE/PASP with invasive PV loop measurements. Hence, our data further validate that the findings of the specific cut-offs applied to the easily-obtainable parameter TAPSE/PASP that reflects RV status, are prognostic in patients with HFrEF and HFpEF and, therefore, might be of clinical use.

In contrast to TAPSE/PASP, the parameter TAPSE alone did not independently predict mortality in our cohort of cardiomyopathy patients. A potential reason for this somewhat-unexpected finding could be the heterogenous nature of our cohort that was without focus on patients with RV alterations. TAPSE/PASP seems to be related to a broader spectrum of RV and LV pathologies than TAPSE, which could explain this difference to TAPSE regarding mortality.

As a potentially-limiting aspect, it must be acknowledged that the clinical data are derived from two prospective observational studies of relatively small sample sizes, which could limit the validity of our findings. 

## 5. Conclusions

Our analysis validates the role of TAPSE/PASP and its cut-off of 0.38 mm/mmHg as echocardiographic parameters of maladaptive RV remodeling that are also associated with mortality. In comparison to TAPSE alone, it shows additional associations to RV fibrosis and maladaptive LV remodeling, which seems to increase its prognostic value in a heterogenous population of patients with ischemic and non-ischemic cardiomyopathy.

## Figures and Tables

**Table 1:** Clinical characteristics of the imaging cohort.

	Data Availability	*n* = 111
Age, y, median (IQR)	111/111	69 (60–76)
Female sex, *n*, (%)	111/111	32 (29)
BMI, kg/m^2^, mean (±SD)	110/111	27 ± 4
BSA, m^2^, mean (±SD)	110/111	1.99 ± 0.22
**Cardiovascular risk factors**		
Hypertension, *n*, (%)	109/111	83 (76)
Diabetes, *n*, (%)	109/111	35 (32)
Insulin, *n*, (%)	109/111	10 (9)
Dyslipidemia, *n*, (%)	105/111	59 (56)
**Clinical history**		
Chronic pulmonary disease, *n*, (%)	109/111	17 (16)
CAD, *n*, (%)	111/111	67 (60)
Prior MI, *n*, (%)	104/111	37 (36)
Prior PCI, *n*, (%)	108/111	41 (38)
Prior CABG, *n*, (%)	110/111	21 (19)
Prior stroke/TIA, *n*, (%)	109/111	17 (16)
Peripheral artery disease, *n*, (%)	109/111	10 (9)
**Symptoms**		
NYHA >/= 3, *n*, (%)	111/111	42 (49)
Syncope, *n*, (%)	109/111	8 (7)
Prior cardiac decompensation, *n*, (%)	47/111	26 (55)
**Echocardiographic findings**		
LVEF, %, median (IQR)	111/111	35 (25–51)
LVEF < 40%, n, (%)	111/111	71 (64)
LVEDd, mm, mean (±SD)	100/111	56 ± 9
LA, mm, median (IQR)	96/111	43 (38–46)
IVSd, mm, median (IQR)	100/111	10 (9–12)
LVPWd, mm, median (IQR)	98/111	10 (9–11)
RVEDd, mm, median (IQR)	96/111	35 (32–40)
TAPSE, mm, mean (±SD)	111/111	18 ± 5
PASP, mmHg, median (IQR)	111/111	39 (30–46)
TAPSE/PASP, mm/mmHg, median (IQR)	111/111	0.46 (0.34–0.66)
AS >/= II, *n*, (%)	107/111	3 (3)
MI >/= II, *n*, (%)	107/111	17 (16)
**Biomarker**		
Creatinine, mmol/L, median (IQR)	105/111	0.92 (0.77–1.26)
eGFR, mL/min/1.73 m², mean (±SD)	102/111	81 ± 28
NT–proBNP, pg/mL, median (IQR)	90/111	1697 (555–4524)
**MRI findings**		
HF, 1/min, median (IQR)	105/111	70 (64–80)
LVEF, %, median (IQR)	107/111	35 (24–50)
LVEDV, mL, median (IQR)	101/111	216 (168–308)
LVEDVI, mL/m^2^, median (IQR)	101/111	108 (86–149)
LVESV, mL, median (IQR)	101/111	75 (47–113)
LVESVI, mL/m^2,^ median (IQR)	101/111	140 (88–232)
LA area, m^2^, mean (± SD)	84/111	28 ± 8
RVEF, %, median (IQR)	101/111	40 (27–51)
RVEDV, mL, median (IQR)	101/111	158 (123–202)
RVEDVI, mL/m^2^, median (IQR)	101/111	78 (70–99)
RV ESVI, mL/m^2^, median (IQR)	101/111	47 (34–67)
RV ESV, ml, median (IQR), ml, median (IQR)	101/111	94 (68–138)
RV SV, mL, median (IQR)	101/111	64 (49–79)
RA area, m^2^, median (IQR)	85/111	26 (23–32)
CI, l/min × m^2^, median (IQR)	101/111	2.6 (2.1–3.2)
LV longitudinal Strain LAX global, %, median (IQR)	44/111	−10 (−14; −7)
RV longitudinal Strain LAX global, %, median (IQR)	44/111	−18 (−24; −10)
T1 time septal, s, median (IQR)	102/111	1165 (1132–1191)
T1 time upper RVIP, s, median (IQR)	96/111	1129 (1102–1181)
T1 time lower RVIP, s, median (IQR)	96/111	1190 (1142–1232)

Abbreviations: BMI body mass index; CAD, coronary artery disease; NYHA, New York Heart Association; PASP pulmonary arterial systolic pressure; PAPmean, mean pulmonary artery pressure; PAWPmean, mean pulmonary artery wedge pressure; TAPSE, tricuspid annular plane systolic excursion; LVEF, left ventricular ejection fraction; RVD, right ventricular diameter; IVSd, diastolic interventricular septum thickness, LVPWd, diastolic left ventricular posterior wall thickness; n.a., not available.

**Table 2:** Correlation analysis of the imaging cohort.

	TAPSE/PASP r	TAPSE r	TAPSE/PASP vs. TAPSE (*p*)
**MRI findings**			
LVEF	0.32 **	0.18	0.04
LV-Masse	−0.29 **	−0.13	0.02
LVEDVI	−0.28 **	−0.02	<0.001
LVESVI	−0.31 **	−0.09	0.001
LA area	−0.34 **	−0.19	0.02
RVEF	0.45 **	0.38 **	0.26
RVEDVI, mL/m^2^	−0.42 **	−0.32 **	0.12
RVESVI, mL/m^2^	−0.52 **	−0.40 **	0.049
RA area	−0.21	−0.15	0.38
RV global longitudinal Strain LAX	−0.51 **	−0.45 **	0.49
LV global longitudinal Strain LAX	−0.40 **	−0.28 *	0.23
T1 septal	−0.22 *	−0.07	0.03
T1 Upper RVIP	−0.37 **	−0.19	0.007
T1 Lower RVIP	−0.32 **	−0.13	0.005
Cardiac Index	−0.02	0.04	0.39
**Biomarkers**			
NT-pro-BNP	−0.70 **	−0.42 **	<0.001
eGFR	0.26 **	0.21 *	0.46

* *p* < 0.05, ** *p* < 0.01.

**Table 3:** Clinical characteristics of the TAPSE/PASP subgroup as part of the validation cohort.

	Data Availability	All	Non Survivors	Survivors	*p*-Value
		***n* = 221**	***n* = 89**	***n* = 132**	
Non-survivors, *n*, (%)	221/221	89 (40)	89 (100)	0 (0)	
Age, y, median (IQR)	221/221	72 (64–78)	73 (68–78)	70 (61–77)	0.03
Female sex, *n*, (%)	221/221	43 (19)	12 (13)	31 (23)	0.08
BMI, kg/m^2^, median (IQR)	221/221	27 (25–31)	27 (25–31)	27 (24–31)	0.73
BSA, m^2^, median (IQR)	221/221	1.99 (1.83–2.15)	2 (1.84–2.11)	1.98 (1.83–2.17)	0.97
**Cardiovascular risk factors**					
Hypertension, *n*, (%)	221/221	179 (81)	73 (82)	106 (80)	0.86
Diabetes, *n*, (%)	221/221	69 (31)	36 (40)	33 (25)	0.02
Dyslipidemia, *n*, (%)	221/221	145 (66)	57 (64)	88 (67)	0.77
**Clinical history**					
CAD, *n*, (%)	221/221	137 (62)	65 (73)	72 (55)	0.01
Prior MI, *n*, (%)	221/221	75 (34)	37 (42)	38 (29)	0.06
Prior PCI, *n*, (%)	221/221	85 (38)	45 (51)	40 (30)	0.003
Prior CABG, *n*, (%)	221/221	47 (21)	25 (28)	22 (17)	0.05
Prior stroke/TIA, *n*, (%)	216/221	27 (13)	15 (17)	12 (9)	0.10
Peripheral artery disease, *n*, (%)	216/221	21 (10)	12 (14)	9 (7)	0.11
**Symptoms**					
NYHA >/= 3, *n*, (%)	221/221	113 (58)	48 (59)	65 (57)	0.58
Syncope, *n*, (%)	200/221	12 (6)	5 (6)	7 (6)	0.98
**Echocardiographic findings**					
LVEF, %, median (IQR)	221/221	30 (25–36)	30 (25–35)	34 (25–40)	0.001
LVEF < 35%, *n*, (%)	221/221	130 (59)	63 (71)	67 (51)	0.003
LVEDd, mm, mean (±SD)	213/221	60 ± 8	62 ± 8	58 ± 8	0.003
LA, mm, median (IQR)	213/221	45 (42–50)	46 (42–50)	45 (42–50)	0.92
IVSd, mm, median (IQR)	206/221	11 (10–12)	11 (10–12)	11 (10–12)	0.93
LVPWd, mm, median (IQR)	191/221	11 (10–12)	11 (9–12)	11 (10–12)	0.22
RVEDd, mm, median (IQR)	185/221	33 (30–37)	33 (30–36)	33 (30–37)	0.65
TAPSE, mm, mean (±SD)	221/221	18 (15–21)	18 (15–21)	17 (15–20)	0.12
PASP, mmHg, median (IQR)	221/221	40 (32–53)	44 (35–56)	38 (31–50)	0.002
TAPSE/PASP, mm/mmHg, median (IQR)	221/221	0.42 (0.32–0.57)	0.38 (0.3–0.51)	0.46 (0.35–0.63)	0.002
AS >= II, *n*, (%)	190/221	26 (14)	15 (19)	11 (10)	0.06
MI >= II, *n*, (%)	219/221	92 (42)	32 (37)	60 (45)	0.17
**Biomarker**					
Creatinine, mmol/L, median (IQR)	220/221	1.02 (0.85–1.3)	1.16 (0.94–1.4)	1 (0.82–1.21)	0.002
GFR, ml/min, median (IQR)	220/221	70 (55–92)	66 (52–82)	74 (59–96)	0.007

## Data Availability

The data presented in this study are available on request from the corresponding author. The data are not publicly available due to privacy restrictions.

## Supplementary Materials

The following are available online at https://www.mdpi.com/article/10.3390/diagnostics11122188/s1.
