# Supplementary material for: Application and Validation of the Tricuspid Annular Plane Systolic Excursion/Systolic Pulmonary Artery Pressure Ratio in Patients with Ischemic and Non-Ischemic Cardiomyopathy
Source: Diagnostics (Basel). 2021 Nov 24;11(12):2188. doi: 10.3390/diagnostics11122188 (PMC8700391; doi:10.3390/diagnostics11122188)
Supplement: Supplementary file 1 [file diagnostics-11-02188-s001.zip › diagnostics-1454872-supplementary.pdf]

A B

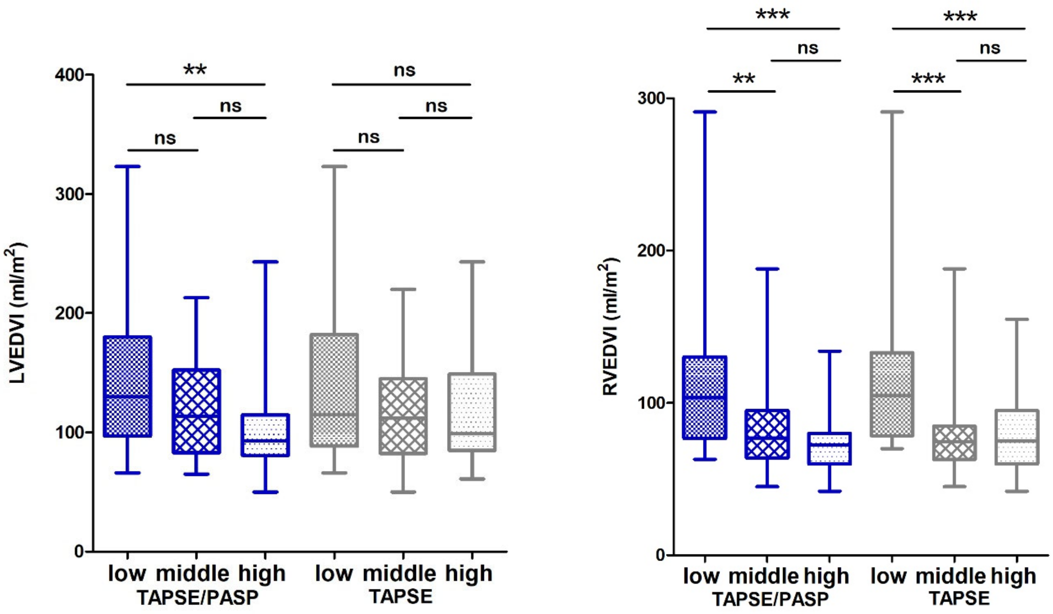

Supplementary Figure S1. Box showing CMR characteristics of TAPSE/PASP and TAPSE tertiles. Shown are values for (A) RVEDVI and (B) LVEDVI divided according to TAPSE/PASP and TAPSE tertiles. Boxes represent median with IQR. ns, not significant, ***p* < 0.01, ****p* < 0.001.

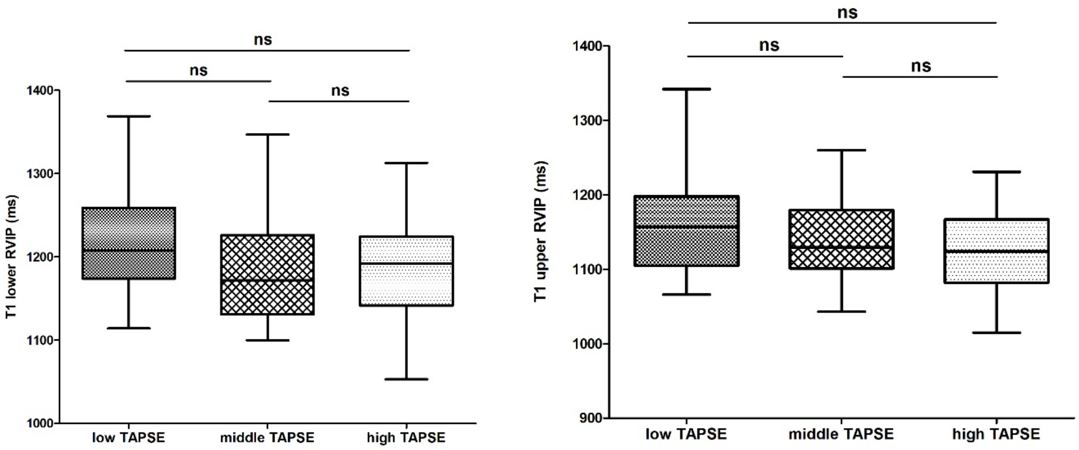

A B

Supplementary Figure S2. Box and scatter plots showing CMR characteristics of TAPSE tertiles. Shown are values for T1 relaxation times in (A) T1 lower RVIP and (B) T1 upper RVIP divided according to TAPSE tertiles. Boxes represent median with IQR. ns, not significant, **p* < 0.05, ***p* < 0.01, ****p* < 0.001.

Supplementary Table S1. Clinical characteristics of TAPSE/PASP tertiles.

	**TAPSE/PASP low <0.38 mm/mmHg**	**TAPSE/PASP middle 0.38–0.63 mm/mmHg**	**TAPSE/PASP High >0.63 mm/mmHg**	***p*-Value**
	**n = 37**	**n = 36**	**n = 38**	
Age, y, median (IQR)	70 (58–76)	69 (63–77)	67 (59–74)	0.69
Female sex, n, (%)	10 (27)	10 (28)	12 (32)	0.90
BMI, kg/m2, mean (± SD)	27 ± 4	28 ± 5	27 ± 4	0.46
BSA, m2, mean (± SD)	1.96 ± 0.22	2.03 ± 0.25	1.99 ± 0.18	0.51
**Cardiovascular risk factors**				
Hypertension, n, (%)	26 (70)	28 (82)	29 (76)	0.48
Diabetes, n, (%)	14 (38)	12 (35)	9 (24)	0.36
Insulin, n, (%)	2 (5)	4 (11)	4 (11)	0.71
Dyslipidemia, n, (%)	21 (58)	22 (67)	16 (44)	0.19
**Clinical history**				
Chronic pulmonary disease, n, (%)	4 (11)	7 (20)	6 (16)	0.61
CAD, n, (%)	23 (62)	21 (58)	23 (61)	0.97
Prior MI, n, (%)	12 (34)	10 (30)	15 (42)	0.64
Prior PCI, n, (%)	15 (41)	11 (33)	15 (39)	0.80
Prior CABG, n, (%)	6 (17)	11 (31)	4 (11)	0.09
Prior stroke/TIA, n, (%)	8 (22)	3 (9)	6 (16)	0.29
Peripheral artery disease, n, (%)	5 (14)	3 (9)	2 (5)	0.46
**Symptoms**				
NYHA >/= 3, n, (%)	16 (53)	18 (58)	8 (33)	0.05
Syncope, n, (%)	2 (6)	4 (11)	2 (5)	0.65
Prior cardiac decompensation, n, (%)	12 (60)	8 (62)	6 (43)	0.61
**Echocardiographic findings**				
LVEF, %, median (IQR)	28 (25–40)	35 (28–50)	50 (35–55)	0.001
LVEF < 35 %, n, (%)	23 (62)	14 (41)	8 (24)	0.002
LVEDd, mm, mean (± SD)	58 ± 9	56 ± 9	54 ± 10	0.30
LA, mm, median (IQR)	44 (43–48)	41 (38–45)	40 (37–44)	0.008
IVSd, mm, median (IQR)	10 (9–12)	10 (9–11)	10 (9–12)	0.83
LVPWd, mm, median (IQR)	10 (10–12)	10 (8–11)	10 (9–11)	0.33
RVEDd, mm, median (IQR)	39 (35–44)	33 (30–38)	34 (31–37)	0.002
TAPSE, mm, mean (± SD)	14 ± 4	18 ± 3	22 ± 3	<0.001
PASP, mmHg, median (IQR)	49 (42–55)	40 (34–45)	28 (25–33)	<0.001
TAPSE/PASP, mm/mmHg, median (IQR)	0.28 (0.22–0.34)	0.46 (0.42–0.53)	0.74 (0.66–0.81)	<0.001
AS >/= II, n, (%)	3 (9)	0 (0)	0 (0)	0.06
MI >/= II, n, (%)	9 (26)	6 (18)	2 (5)	0.047
**Biomarker**				
Creatinine, mmol/L, median (IQR)	1.14 (0.86–1.44)	0.9 (0.79–1.27)	0.84 (0.7–1.02)	0.01
eGFR, ml/min/1.73 m², mean (± SD)	72 ± 28	80 ± 26	89 ± 29	0.08
NT-proBNP, pg/ml, median (IQR)	5829 (2084–9318)	1933 (807–3950)	542 (341–1376)	<0.001
**MRI findings**				
HF, 1/min, median (IQR)	71 (65–86)	70 (66–83)	70 (60–74)	0.26
LVEF, %, median (IQR)	27 (20–38)	30 (24–50)	47 (31–59)	0.001
LVEDV, ml, median (IQR)	246 (186–343)	230 (162–328)	177 (162–221)	0.01
LVEDVI, ml/m2, median (IQR)	130 (99–168)	114 (86–152)	92 (80–109)	0.004
LVESV, ml, median (IQR)	172 (120–262)	158 (90–235)	102 (74–150)	0.003
LVESVI, ml/m2, median (IQR)	90 (65–131)	82 (46–111)	51 (36–75)	0.001
LA area, m2, mean (± SD)	31 ± 7	28 ± 9	26 ± 7	0.02
RVEF, %, median (IQR)	32 (19–40)	44 (30–50)	50 (42–58)	<0.001
RVEDV, ml, median (IQR)	192 (148–248)	170 (141–187)	141 (115–162)	<0.001
RVEDVI, ml/m2, median (IQR)	104 (77–130)	77 (64–95)	73 (60–80)	<0.001
RV ESVI, ml/m2, median (IQR)	66 (52–99)	47 (33–60)	34 (26–44)	<0.001
RV ESV, ml, median (IQR), ml, median (IQR)	128 (92.5–188.25)	97.5 (71–137.75)	70 (55–92)	<0.001
RV SV, ml, median (IQR)	61.5 (47.25–78)	58.5 (49–81.75)	68 (53.5–77.5)	0.72
RA area, m2, median (IQR)	29 (24–36)	25 (24–30)	25 (23–29)	0.10
CI, l/min*m2, median (IQR)	2.7 (2.1–3.2)	2.5 (2.2–2.8)	2.6 (2.1–3.2)	0.74
LV longitudinal Strain LAX global, %, median (IQR)	−8 (−11; −5)	−8 (−14; −7)	−14 (−19; −10)	0.001
RV longitudinal Strain LAX global, %, median (IQR)	−15 (−19; −8)	−16 (−20; −8)	−25 (−26; −24)	<0.001
T1 time septal, s, median (IQR)	1178 (1150–1202)	1158 (1121–1190)	1144 (1126–1183)	0.03
T1 time upper RVIP, s, median (IQR)	1170 (1120–1219)	1136 (1105–1199)	1117 (1073–1130)	<0.001

T1 time lower RVIP, s, median (IQR)	1208 (1174–1259)	1172 (1131–1226)	1192 (1142–1225)	0.001

Supplementary Table S2. Multivariable regression analysis of predictors of 10-years mortality.

**Model 1:**			
**Variable**	***p*-value**	**Odds Ratio**	**95% CI**
**PASP**	0.21	1.01	0.99–1.04
**LVEDd**	0.01	1.06	1.01–1.11
**LVEF**	0.17	0.97	0.93–1.01
**eGFR**	0.11	0.99	0.98–1.00
**Diabetes**	0.13	1.66	0.86–3.19
**Prior PCI**	0.01	2.22	1.20–4.13
**Age**	0.08	1.03	0.99–1.07
**Model 2:**			
**Variable**	***p* value**	**Odds Ratio**	**95% CI**
**TAPSE/PASP**	0.02	0.12	0.02–0.71
**LVEDd**	0.02	1.05	1.01–1.10
**LVEF**	0.48	0.98	0.94–1.03
**eGFR**	0.05	0.99	0.98–1.01
**Diabetes**	0.11	1.71	0.89–3.28
**Prior PCI**	0.01	2.24	1.21–4.16
